# Activity and structural analysis of GRL-117C: a novel small molecule CCR5 inhibitor active against R5-tropic HIV-1s

**DOI:** 10.1038/s41598-019-41080-w

**Published:** 2019-03-18

**Authors:** Hirotomo Nakata, Kenji Maeda, Debananda Das, Simon B. Chang, Kouki Matsuda, Kalapala Venkateswara Rao, Shigeyoshi Harada, Kazuhisa Yoshimura, Arun K. Ghosh, Hiroaki Mitsuya

**Affiliations:** 1 Experimental Retrovirology Section, HIV and AIDS Malignancy Branch, National Cancer Institute, National Institutes of Health, Bethesda, Maryland 20892-1868 USA; 2 Departments of Hematology and Infectious Diseases, Kumamoto University Graduate School of Medical and Pharmaceutical Sciences, Kumamoto, 860-8556 Japan; 3 National Center for Global Health and Medicine Research Institute, Tokyo, 162-8655 Japan; 4 Department of Chemistry and department of Medicinal Chemistry and Molecular pharmacology, Purdue University, West Lafayette, Indiana, USA; 5 AIDS Research Centre, National Institute of Infectious Diseases, Tokyo, 162-8640 Japan

## Abstract

CCR5 is a member of the G-protein coupled receptor family that serves as an essential co-receptor for cellular entry of R5-tropic HIV-1, and is a validated target for therapeutics against HIV-1 infections. In the present study, we designed and synthesized a series of novel small CCR5 inhibitors and evaluated their antiviral activity. GRL-117C inhibited the replication of wild-type R5-HIV-1 with a sub-nanomolar IC_50_ value. These derivatives retained activity against vicriviroc-resistant HIV-1s, but did not show activity against maraviroc (MVC)-resistant HIV-1. Structural modeling indicated that the binding of compounds to CCR5 occurs in the hydrophobic cavity of CCR5 under the second extracellular loop, and amino acids critical for their binding were almost similar with those of MVC, which explains viral cross-resistance with MVC. On the other hand, one derivative, GRL-10018C, less potent against HIV-1, but more potent in inhibiting CC-chemokine binding, occupied the upper region of the binding cavity with its *bis*-THF moiety, presumably causing greater steric hindrance with CC-chemokines. Recent studies have shown additional unique features of certain CCR5 inhibitors such as immunomodulating properties and HIV-1 latency reversal properties, and thus, continuous efforts in developing new CCR5 inhibitors with unique binding profiles is necessary.

## Introduction

Small molecule CCR5 inhibitors cause allosteric changes in the conformation of CCR5, which is a critical coreceptor involved in the binding of the gp120/CD4 complex, allowing CCR5 inhibitors to block the fusion between cellular and viral membranes, and inhibit the entry of HIV-1^1–3^. As their names suggest, entry inhibitors block entry of HIV-1 into the cells, and are distinct from other classes of HIV-1 inhibitors, such as reverse transcriptase inhibitors (RTIs), integrase inhibitors (INIs), and protease inhibitors (PIs), all of which block HIV-1 replication following its entry into the cell^1,2^. Therefore, CCR5 inhibitors are considered to be effective against wild-type HIV-1 species as well as HIV-1 variants that are resistant to existing classes of anti-retroviral drugs including most recent INIs. In addition, if properly combined with other classes of anti-retroviral drugs, CCR5 inhibitors are expected to exert synergistic effects^4,5^.

As for drug-resistance against CCR5 inhibitors, a clinical trial (MERIT study) for maraviroc (MVC) showed that the level of MVC resistance was low, and that the virologic failure observed was mainly caused by the emergence of pre-existing X4-HIV-1 that was not detected by the tropism assay^6^. However, some groups have reported that CCR5 inhibitor-resistant viruses can be developed *in vitro*^7–10^. Trkola *et al*., and Marozsan *et al*., reported the generation of HIV-1 escape mutants for AD101 (experimental CCR5 inhibitor) and SCH-D (vicriviroc or VVC). They also found that such escape mutant viruses did not use CXCR4, but instead gained the ability to use CCR5 in a CCR5 inhibitor-insensitive manner^7,8,11^. Westby *et al*., reported the selection of an MVC-resistant virus with mutations in the V3 loop of HIV-gp120 that acquired resistance without tropism shift from R5 to X4^9^. These results suggest that long-term usage of MVC may result in the emergence of MVC-resistant R5 HIV-1s.

Currently, MVC is the only CCR5 inhibitor in clinical use. MVC was first approved by the FDA as the first-in-class CCR5 inhibitor in 2007. However, MVC has some disadvantages as an anti-HIV drug. One limitation of MVC is that it needs to be dosed twice daily, whereas other recent HIV-1 drugs such as tenofovir (RTI), dolutegravir (INI), and darunavir (PI) need to be administered once a day only. In addition, patients must be examined for viral tropism in their body before starting a treatment with MVC. Thus, MVC is currently not often used in clinic compared to other conventional anti-HIV drugs.

In the meantime, recent studies have shown a possibility of the usage of CCR5 inhibitors besides that of a conventional anti-HIV drug. Another potent CCR5 inhibitor, cenicriviroc (TBR-652 or CVC)^12^ was reported to interact with CCR2 as well, which is associated with inflammation-related diseases, and is expected to be a potential inflammatory mediator^12^. In fact, Krenkel *et al*., reported therapeutic effects of inhibiting monocyte infiltration in nonalcoholic steatohepatitis (NASH) models by using CVC to inhibit the recruitment of Kupffer cells and monocyte-derived cells^13^. On the other hand, the administration of MVC also reportedly results in the increase of CD4^+^ T-cell counts in the blood, which was considered a possible advantage that may contribute to increased immunological functions^14^. Moreover, another group recently reported that MVC reversed HIV-1 latency *in vitro* alone or in combination with a PKC agonist^15^, suggesting a possibility of the utility of MVC as a latency-reversing agent. However, such effects of CCR5 (CCR2) inhibitors on chemokine-induced cellular/immunological function are considered to be very complicated and exact mechanisms underlying such phenomenon are not known. Thus, the development of new CCR5 inhibitors with favorable pharmacokinetics (once-daily regimens), unique binding profiles to CCR5, and unique immunological features is desired.

In this study, we report several novel small molecule CCR5 inhibitors that demonstrate potent anti-R5-HIV-1 activity. We also elucidated their binding mode and interactions with CCR5, and compared their biological/structural characteristics with that of MVC.

## Results

### Activity of GRL-117C and its derivatives against R5 HIV-1

We designed and synthesized small molecule compounds as candidates for novel CCR5 inhibitors, and identified several compounds that have potent activity against wild type R5-HIV-1. GRL-117C exerted potent activity against R5-HIV-1_Ba-L_ with a sub-nanomolar IC_50_ value in the MAGI assay using MAGI/CCR5 cells. The potency (IC_50_ values) of GRL-117C was comparable to that of MVC, as was determined by both the MAGI assay (0.6 nM vs. 0.7 nM) and the p24 assay with PBMCs (8.1 nM vs. 4.5 nM). APL^16,17^ demonstrated similar or slightly more potent activity than MVC, and its IC_50_ values were 0.2 nM and 2.6 nM for the MAGI and p24 assays, respectively. The other GRL-compounds, GRL-10007C and GRL-10018C, also demonstrated strong activity against HIV-1_Ba-L_ in the MAGI assay (IC_50_: 1.4 nM and 2.9 nM, respectively). These compounds were found to be more potent compared to the two previously published experimental CCR5 inhibitors, SCH-C and TAK-779, but were less effective than MVC and APL (Table 1). Two drug-naïve clinical R5-HIV-1 strains, CC1/85 cl.6 and cl.7, were also used in the assays^7,8^. All the compounds tested in this study showed similar effectiveness against the CC1/85 clinical strains compared to HIV-1_Ba-L_ (Table 1). We have previously observed that the IC_50_ value(s) of CCR5 inhibitors in MAGI assays^18^ tended to be lower compared to those obtained via the p24 assays in PBMCs^16,19^. In this study, we also observed the same trend. For example, the IC_50_ value of GRL-117C for the MAGI assay was 0.6 nM, but was 8.1 nM for the p24 assay (HIV-1_Ba-L_) (Table 1).

**Table 1:** Activity of CCR5 inhibitors against HIV-1s, including CCR5 inhibitor-resistant HIV-1s.

Virus	IC_50_ [IC_90_] (nM)						
	MVC	SCH-C	APL	TAK-779	GRL-117C	GRL-10007C	GRL-10018C
Ba-L (MAGI)^a^	0.7 [6.4]	4.8 [15.9]	0.2 [4.9]	15.7 [88.1]	0.6 [8.9]	1.4 [9.5]	2.9 [27.5]
Ba-L (PBMCs)^b^	4.5 [21.6]	13.9 [97.2]	2.6 [13.4]	31.6 [187]	8.1 [48.3]	16.9 [126.5]	18.7 [154.7]
CC1/85 cl.6^c^	1.7 [n.d.^d^]	5.2 [n.d.^d^]	1.5 [n.d.^d^]	22.7 [n.d.^d^]	n.d.^d^ [n.d.^d^]	12.5 [n.d.^d^]	n.d.^d^ [n.d.^d^]
CC1/85 cl.7^c^	2.5 [20.6]	6.1 [67.7]	1.8 [11.3]	28.3 [101]	3.5 [28.9]	15.8 [80.3]	4.9 [34.9]
CC101.19 cl.7	36.9 [486]	>1000 [>1000]	16.3 [200]	400 [>1000]	32.5 [296]	41.1 [472]	52.5 [553]
(AD101 resistant)	(×15)^e^	(>×164)	(×9.1)	(×14)	(×9.3)	(×2.6)	(×11)
D1/85.16 cl.23	26.5 [376]	412 [>1000]	8.6 [98.6]	166 [747]	29.8 [415]	56.9 [536]	61.3 [642]
(VVC resistant)	(×11)^e^	(×68)	(×4.7)	(×5.9)	(×8.5)	(×3.6)	(×12.5)

^a^IC_50_/[IC_90_] numbers were obtained by MAGI assay using MAGI cells and R5-HIV-1_BaL_.

^b^IC_50_/[IC_90_] numbers were obtained by p24 assay using PBMCs and R5-HIV-1_BaL_.

^c^Drug naïve R5-HIV-1 clinical strains.

^d^not done.

^e^Parentheses indicate fold increases of IC_50_s compared to wild type (CC1/85 cl.7).

### Activity of CCR5 inhibitors against transmitter/founder (T/F) HIV-1s

We also examined the activity of GRL-compounds against Transmitter/founder (T/F) HIV-1 viruses. T/F viruses are involved in the initial infection. It is considered that T/F viruses virtually always use CCR5 rather than CXCR4 and infect T cells but not macrophages because high level of CD4 is needed to mediate virus entry for the initial transmission^20–24^. Thus, CCR5 inhibitors are expected to be active against such T/F viruses. We obtained four HIV-1 T/F infectious clones. We determined the antiviral activity of GRL-117C, GRL-10007C and GRL-10018C on these HIV-1 clones using MAGI assay. We also determined the activity of Maraviroc and APL against these viruses. As shown in Table 2, GRL-CCR5 inhibitors, especially GRL-117C, exerted potent activity against all four T/F viruses. Maraviroc and APL were also highly potent. The IC_50_ values of GRL-117C were 1.9–3.2 nM, and were substantially similar to the activity of MVC (1.7–2.3 nM) against these cells (Table 2).

**Table 2:** Activity of CCR5 inhibitors against transmitted/founder (T/F) HIV-1s.

HIV-1 infectious molecular clones	IC_50_ (nM)^a^				
	MVC	APL	GRL-117C	GRL-10007C	GRL-10018C
pCH040.c/2625	1.7 ± 0.1^b^	0.7 ± 0.2	2.0 ± 0.6	3.1 ± 0.3	7.2 ± 1.4
pCH0.6.c/2633	2.3 ± 0.3	1.9 ± 0.2	1.9 ± 0.3	5.3 ± 3.4	8.8 ± 4.2
pTHRO.c/2626	2.2 ± 0.8	0.9 ± 0.1	2.1 ± 0.2	3.2 ± 0.4	3.9 ± 1.1
pCH058.c/2960	1.9 ± 0.1	1.7 ± 0.2	3.2 ± 1.0	6.1 ± 3.2	8.0 ± 2.3

^a^IC_50_ numbers were obtained by MAGI assay using MAGI cells.

^b^Data are expressed as mean ± SD.

### Activity of CCR5 inhibitors against CCR5 inhibitor-resistant HIV-1s

**Figure 1:**
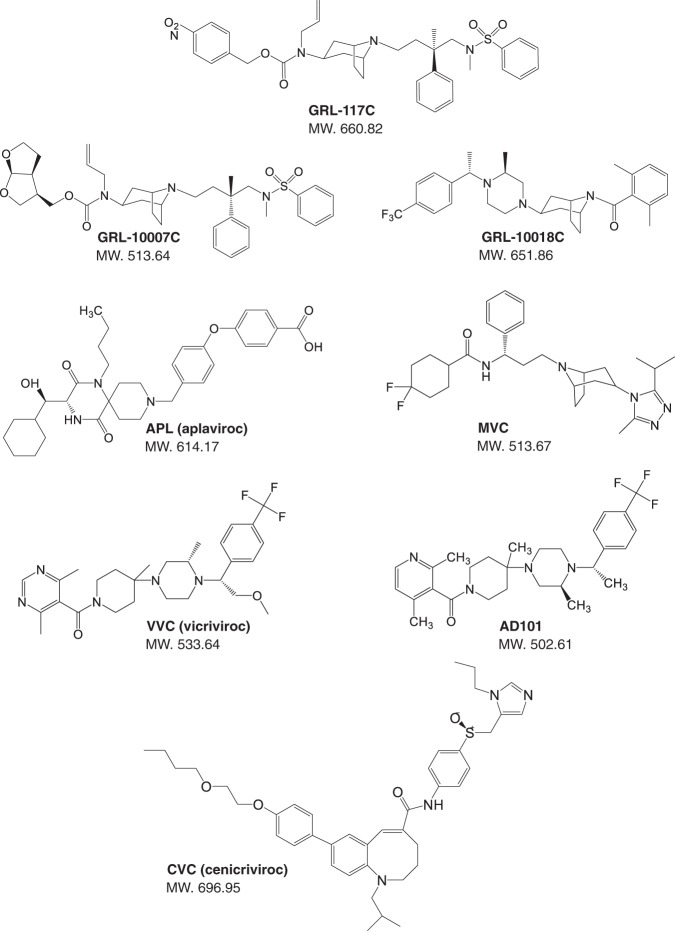
Structures of GRL-CCR5 inhibitors (GRL-117C, GRL-10007C, and GRL-10018C) and APL (aplaviroc), MVC (maraviroc), VVC (vicriviroc), AD101, and CVC (cenicriviroc).

We selected three compounds (GRL-117C, GRL-10007C, and GRL-10018C) (Fig. 1) for further testing. In a previous study, Trkola *et al*., reported the generation of an escape mutant HIV-1 for AD101 (experimental CCR5 inhibitor), and found that this mutant did not use CXCR4, but instead gained the ability to use CCR5 in an AD101-insensitive manner^7^. Subsequently, Marozsan *et al*., described the generation of escape mutants under the selection pressure of VVC *in vitro*^8^. Both escape mutants were fully resistant against AD101 and VVC^7,8^. For the current study, AD101- and VVC-resistant HIV clones were provided by Dr. John P. Moore of Cornell University. As shown in Table 1, CC101.19 (AD101-resistant) was approximately 150-fold more resistant to SCH-C (IC_50_: >1,000 nM) compared to its corresponding CCR5 inhibitor-sensitive viruses, CC1/85 (cl.6 and cl7, IC_50_: 5.2 and 6.1 nM, respectively). On the other hand, resistance against other CCR5 inhibitors, including MVC, APL, and GRL-compounds, were relatively lower in comparison; fold resistance ranged from 2.6- to 15-fold. The VVC-resistant virus (D1/85.16) also showed high resistance against SCH-C (68-fold), but remained susceptible to all other drugs to some extent (fold resistance: 3.6-fold to 12.5-fold). GRL-117C exhibited slightly decreased activity against AD101- and VVC-resistant viruses (fold resistance: 9.3- and 8.5-fold, respectively), however, its IC_50_ numbers remained less than 40 nM. Interestingly, GRL-10007C, which was less reactive than GRL-117C against wild type R5-HIV-1, maintained its activity against AD101- and VVC-resistant viruses, showing IC_50_ values of 41.1 nM (2.6-fold) and 56.9 nM (3.6-fold) (Table 1). This result suggested that the resistance profiles of SCH-C and its associated drugs (VVC and AD101) differ drastically from those of MVC, APL, and GRL-derivatives. GRL-10007C, which induced the least resistance in these viruses, may have a unique resistance profile among the CCR5 inhibitors tested in this study.

**Figure 2:**
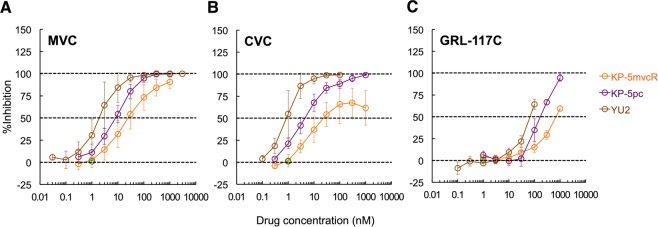
Inhibitory effect on HIV-1 replication in TZM-bl cells. Percent inhibition of (**A**) MVC, (**B**) CVC, and (**C**) GRL-117C on HIV-1 replication is shown. Data are shown as means ± s.d. of three independent experiments.

We then wanted to determine if these compounds are effective against HIV-1s carrying MVC-resistance-associated substitutions. As shown in Fig. 2 and Table 3, activity of GRL-117C was reduced when used against the highly MVC-resistant virus (HIV-1_KP-5mvcR_) (IC_50_: 686 nM). However, GRL-117C also demonstrated decreased activity against a drug-naïve HIV-1 clinical strain (HIV-1_KP-5pc_) as compared to the laboratory HIV-1 strain (HIV-1_YU2_) (Table 3). Therefore, while the fold change of IC_50_ values for GRL-117C was only 4.8 between HIV-1_KP-5pc_ and HIV-1_KP-5mvcR_, we concluded that GRL-117C had cross-resistance with MVC, because its IC_50_ value against HIV-1_KP-5mvcR_ (686 nM) was more than 10-fold greater than that of MVC (41 nM) (Table 3). The other derivatives, GRL-10007C and GRL-10018C also failed to demonstrate activity against HIV-1_KP-5mvcR_ (data not shown). It is of note that the activity of CVC against HIV-1_KP-5mvcR_ was substantially decreased compared to that of wild type [IC_50_: 260 nM vs. 4.1 nM (×63-fold)], indicating that it also has cross-resistance with MVC (Table 3).

**Table 3:** Activity of CCR5 inhibitors against HIV-1s, including MVC-resistant HIV-1.

Virus	IC_50_ [IC_90_] values (nM)^a^		
	MVC	CVC	GRL-117C
YU2^b^	3.5 ± 2.5 [15 ± 15]	1.1 ± 0.7 [6.2 ± 5.7]	68 ± 8.8 [>100]
KP-5pc^b^	8.5 ± 3.6 [81 ± 41]	4.9 ± 2.2 [105 ± 62]	160 ± 30 [767 ± 26]
KP-5mvcR^c^	36 ± 28 [236 ± 146]	260 ± 352 [>300]	686 ± 89 [>100]

^a^IC_50_ [IC_90_] numbers were obtained via the TZM-bl assay. Data are expressed as mean ± SD.

^b^Drug-sensitive HIV-1 strains.

^c^MVC-resistant HIV-1 strain.

### GRL-CCR5 inhibitors inhibit the binding of CC-chemokines to CCR5

**Figure 3:**
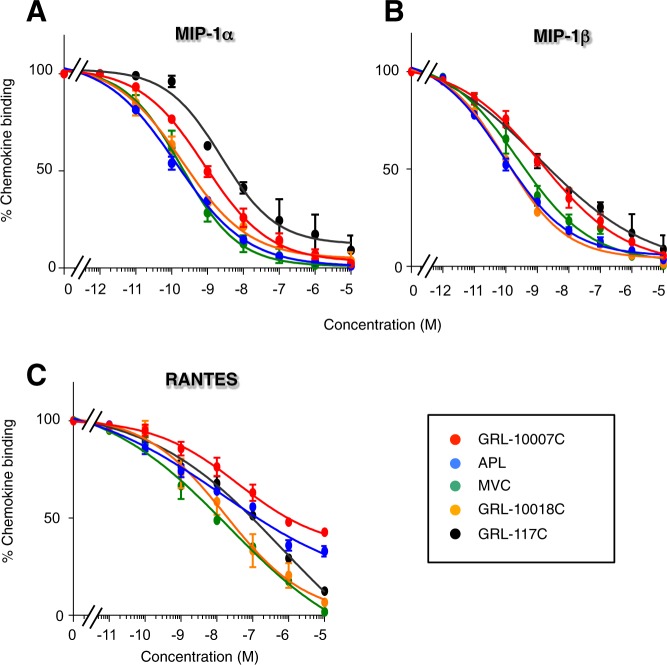
Inhibition of ^125^I-labeled CC-chemokine binding to CCR5 by CCR5 inhibitors. CCR5^+^ CHO cells were incubated with (**A**) ^125^I-MIP-1α, (**B**) ^125^I-MIP-1β, and (**C**) ^125^I-RANTES (200 ng/ml) in the presence or absence of varying concentrations of CCR5 inhibitors. Data are shown as means ± s.d. of three independent experiments.

In order to determine whether GRL derivatives block the binding of CC-chemokines to CCR5, we conducted a CC-chemokine binding inhibition assay using ^125^I-labeled CC-chemokines (^125^I-RANTES, ^125^I-MIP-1α, and ^125^I-MIP-1β) and CCR5 expressing cells. All the CCR5 inhibitors tested (GRL-117C, GRL-10007C, GRL-10018C, MVC, and APL) blocked the binding of ^125^I MIP-1α to CCR5, and their EC_50_ values ranged from 0.1–4.3 nM. Similar results were observed for MIP-1β binding (EC_50_ range: 0.2–2.5 nM) (Fig. 3 and Table 4). Results demonstrated that MVC, APL, and GRL-10018C exert stronger inhibitory effects on MIP-1α and MIP-1β binding compared to GRL-117C and GRL-10007C. In contrast, APL, GRL-117C, and GRL-10007C only moderately blocked the binding of RANTES; their EC_50_ values were 156, 121, and 628 nM, respectively (Table 4), and binding of ^125^I-RANTES remained at more than 40% even in the presence of 1 μM of GRL-10007C (Fig. 3). We have previously reported that APL does not effectively inhibit the binding and function of RANTES, even though it binds to CCR5^16^. It is possible that GRL-10007C and GRL-117C also have similar profiles as APL in terms of their role in CC-chemokine to CCR5 binding.

**Table 4:** Inhibition of CC-chemokine binding by CCR5 inhibitors.

Compound	EC_50_ values (nM)^a^		
	MIP-1α	MIP-1β	RANTES
GRL-117C	4.3	2.0	121
GRL-10007C	1.1	2.5	628
GRL-10018C	0.3	0.4	17
MVC	0.1	0.5	8.4
APL	0.2	0.2	156

^a^EC_50_ values of cytosolic Ca^2+^ mobilization (Ca^2+^ flux) were determined by comparison with the Ca^2+^ flux level in drug-free control samples.

### Determination of the binding site and binding profile of GRL-117C via structural analysis

**Figure 4:**
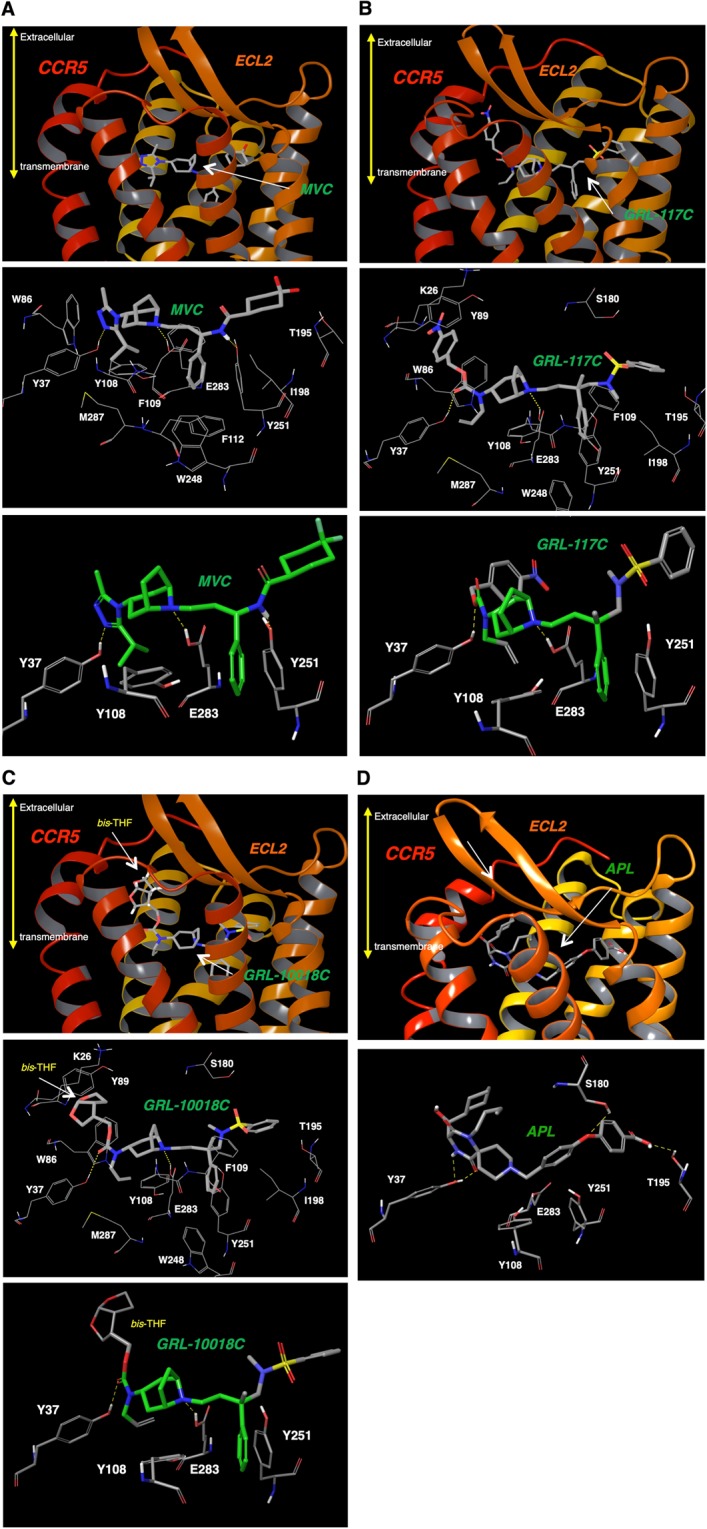
Binding pocket of CCR5. A side view of CCR5 with the bound CCR5 inhibitor is shown. (**A**) MVC, (**B**) GRL-117C, (**C**) GRL-10018C, and (**D**) APL in the binding pocket of CCR5 are illustrated. (Upper) The transmembrane (TM) domains and the second extracellular loop (ECL2) are indicated. (Center) Detailed interactions between a CCR5 inhibitor and CCR5 residues are shown. The *bis*-THF moiety of GRL-10018C (**C**) locates upper region of the binding cavity. APL (**D**) has hydrogen-bond interactions with Y37, S180 and T195 (Bottom) Interactions of each CCR5 inhibitor (MVC, GRL-117C, and GRL-10018C) with Y37, E283, and Y251. Yellow dot: hydrogen-bonding.

Three-dimensional models of human CCR5-CCR5 inhibitor complexes were defined using the crystal structure of CCR5-MVC as the template (PDB ID: 4MBS)^25^. As was previously reported by Tan *et al*., MVC was found to be lodged in the bottom of the largest pocket at the binding site, which was defined by residues from helices 1, 2, 3, 5, 6, and 7 (Fig. 4A and ref.^25^). It was observed that MVC forms hydrogen bonds with Tyr-37, Tyr-251, and Glu-283, and its phenyl group reaches deep into the pocket to form hydrophobic interactions with aromatic residues such as Tyr-108, Trp-248, and Tyr-251 (Fig. 4A and ref.^25^). As shown in Fig. 4B, GRL-117C also binds to the same binding cavity, and similar to MVC, GRL-117C forms hydrogen bonds with Tyr-37 and Glu-283, but not with Tyr-251. Binding models of GRL-10018C with CCR5 also showed formation of hydrogen bonds between GRL-10018C and Tyr-37 and Glu-283 (Fig. 4C). On the other hand, overall, APL binds in the same active site cavity as previously reported^26,27^. There are polar interactions with Tyr-37, Ser-180 and Thr-195. Tyr-108, Tyr-251 and Glu-283 are part of the binding pocket and form non-polar interactions with APL (Fig. 4D)

As described, GRL compounds (117C and 10018C) and MVC have hydrogen-bond interactions with Tyr-37 and Glu-283 (Fig. 4A–C). MVC also has a hydrogen-bonding with Tyr-251 (Fig. 4A). On the other hand, for GRL-117C and 10018 C, there are weak non-polar interactions and very weak pi-pi interactions between the phenyl groups of GRL-compounds and Tyr-251 (Fig. 4B,C), thus making slightly different binding profiles of GRL-compounds to the CCR5 binding cavity comparing to that of MVC. As was also described in the previous section, GRL-10018C exhibited more potent inhibitory effects on the binding of the three chemokines as compared to GRL-117C and GRL-10007C (Fig. 3 and Table 4). This may be due to the fact that GRL-10018C possesses a *bis-*THF structure. The bulky rings occupying the upper region of the binding cavity under ECL2 cause steric hindrance with CC-chemokine when it binds to CCR5. However, the *bis-*THF structure does not affect the interaction between gp120 with CCR5. Moreover, as was previously reported by Tan *et al*., the phenyl group of MVC form hydrophobic interactions with Trp-248, thus preventing its activation-related motion (Fig. 4A)^25^. Similarly, GRL-117C and GRL-10018 also have a phenyl ring in the center of their structure that forms hydrophobic interactions with Trp-248; it is therefore possible that GRL-derivatives also prevent activation-related motion of CCR5.

## Discussion

In the present study, we investigated a series of novel small CCR5 inhibitors that are active against R5-HIV-1, including CCR5 inhibitor-resistant HIV-1s. While proof-of-principle studies have shown that CCR5 inhibitors can be effective against HIV-1 infections and AIDS, the use of CCR5 inhibitors still raises some concerns. One issue is the acquisition of resistance against CCR5 inhibitors. Previous studies have suggested two possible mechanisms by which resistance against CCR5 inhibitors is acquired: i) tropism change (X4-HIV-1 becomes predominant in the patient body), and ii) emergence R5-HIV-1 variants that can utilize “drug-bound” CCR5 for viral entry into the cell^2,9–11^. In MVC-resistant virus, Westby *et al*., found that an isolate had A316T and I323V mutations in the V3 loop of gp120. In addition, the resistant virus strain exhibited a unique profile; while a decrease in maximal inhibition with MVC was observed, its IC_50_ value did not shift substantially^9^. It is possible that this may be due to the noncompetitive profiles of MVC and VVC inhibition. Even in the presence of CCR5 inhibitors, the resistant virus was able to bind to the CCR5-drug complex (drug-bound CCR5)^9–11^. In the present study, we also observed drastic increase of IC_90_ number of MVC against MVC-resistant strain (Table 2), and it is possibly due to “drug-bound CCR5” utilization mechanism (Fig. 2). We also demonstrated that activity of GRL-117C was reduced against highly MVC-resistant virus (HIV-1_KP-5mvcR_) (IC_50_: 686 nM) (Table 3). However, GRL-117C also showed reduced activity against the drug-naïve HIV-1 clinical strain (HIV-1_KP-5pc_) as compared to the laboratory HIV-1 strain (HIV-1_YU2_) (Table 3). Taken together, it is predicted that the activity/resistance profile of GRL-117C is basically the same but slightly different from that of MVC, presumably because of their slightly different binding profiles to CCR5, as shown in Fig. 4. On the other hand, Westby *et al*., reported that MVC-resistant recombinant viruses retained sensitivity to APL, another experimental CCR5 inhibitor^9,16^. Aside from the residues in the deeper TM domains (e.g., Glu-283), APL also interacts with residues in ECL2 (e.g., Cys-178, Ser-180, and Lys-191)^26^, resulting in a significantly different binding mode to that of MVC (Fig. 4A). This may imply that optimization of recent CCR5 inhibitors with modified binding modes similar/close to APL may result in better activity against MVC-resistant viruses.

As discussed above, the acquisition of drug-resistance against CCR5 inhibitors in viruses is a serious problem. However, recent studies have reported that CCR5 inhibitor-resistant viruses are sensitive to neutralization via the V3 and CD4i epitopes^10,28^. In addition, Yoshimura *et al*. suggested that the neutralizing epitopes on MVC-resistant viruses with a greater number of mutations could be more efficiently exposed for such mAbs^10^. These findings suggest that CCR5 inhibitors may be used to enhance the effect of mAb neutralization or antibody-dependent cell-mediated cytotoxicity (ADCC) activity^10^.

The binding modes of the inhibitors presented here were determined by docking to a crystal structure of CCR5 (PDB ID: 4MBS)^25^ (please see Methods section for details). We carried out validation studies to make sure Glide, the docking program used here, is able to generate accurate docking poses by docking Maraviroc back into the crystal structure of CCR5. In fact, there is no difference in the binding mode of MVC obtained by our docking protocol from the binding mode observed in the crystal structure. GRL-117C, GRL-10018C, and GRL-10007C share a common substructure with MVC. This substructure occupies the same region of the binding cavity for both MVC as well as the GRL-inhibitors studied here. Moreover, the phenyl group of Tyr-251 has non-polar interactions with a phenyl moiety of GRL-117C, GRL-10018C, and GRL-10007C and MVC (Fig. 4A–C).

In general, binding studies on mutant residues can give very useful information on binding interactions of specific residues with inhibitors. However, Tyr-108, Tyr-251 and Glu-283 present in the binding pocket of CCR5 pose challenges. Our previous studies^26,27^ have shown the importance of Tyr-251 in maintaining the shape of the binding cavity. Tyr-251 and Glu-283 are also important in the interaction of CCR5 with chemokines and gp120. Tyr-251 and Glu-283 form polar interactions with each other and are responsible for maintaining the optimum shape of the cavity^25–27^. Mutagenesis is very likely to disrupt this optimal shape, and interfere with binding. The crystal structure of CCR5-MVC showed the importance of Tyr-251 and Glu-283 in the binding pocket as well as the hydrophobic interaction by the phenyl group of MVC with Tyr-251^25^. Thus, we consider that the modeling of GRL compounds to CCR5 using MVC-CCR5 co-crystal as a template shown in this study is reliable enough without mutagenesis data.

As CCR5 is an important co-receptor for the cellular entry of HIV, it is considered that the prophylactic use of CCR5 inhibitors is a potential strategy to prevent HIV transmission. Transmitter/founder (T/F) viruses are involved in the initial infection, and virtually always use CCR5 rather than CXCR4 and infect T cells but not macrophages. In addition, Parker *et al*. reported that the chronically-infected viruses had envelopes that exhibited partial resistance to MVC (52%), while, only 15% of T/F viruses exhibited that property^23^, suggesting higher sensitivity of T/F viruses to MVC. In fact, the data presented in this study also showed the potent activity of CCR5 inhibitors, including GRL-CCR5 inhibitors, against T/F viruses (Table 2).

To develop an effective prevention strategy using CCR5 inhibitors, Herrera *et al*. performed *ex-vivo* preclinical evaluation using colorectal tissue explants to determine the efficacy of MVC in combination with reverse transcriptase inhibitors (RTIs) and found that the drug combination(s) inhibited HIV-1 transmission at viral entry^29^. Brocca-Cofano *et al*. examined the effect of MVC on simian immunodeficiency virus (SIV) transmission to infant macaques. However, the authors found that MVC had only a marginal effect in inhibiting the transmission of a T/F variant and a minimal impact on the postinfection delay of viremia following oral SIV infection^30^. The result suggests that more studies are needed to develop optimal prevention strategies using entry inhibitors. It is also possible that the partial effect of MVC is due to a limited efficacy of the drug, and more effective novel CCR5 inhibitors may have a better effect in preventing HIV transmission^30,31^.

Another concern for the use of CCR5 inhibitors is the long-term safety associated with blockage of CCR5, a receptor whose function is not yet fully understood in healthy individuals. We have previously shown that APL only partially blocks the CCR5-dependent binding and signal induction of RANTES^16^. In the present study, we showed that GRL-117C and GRL-10007C also only moderately blocked the binding of RANTES (Table 4), suggesting that they may have similar profiles as APL in terms of CC-chemokine binding inhibition. On the other hand, GRL-10018C showed quite different profile of chemokine binding inhibition (Fig. 3).

CVC, another CCR5 inhibitor currently under clinical trials^32^, was recently reported to interact with CCR2 as well, and is expected to be a potential inflammation mediator^12^. Moreover, MVC is also known to increase CD4^+^ T-cell counts in the blood^14^. This may suggest a possibility that some of the GRL-CCR5 inhibitors (e.g. GRL-10018C) may be optimized as a new CCR5 inhibitor that has some additional usage such as CVC. However, such effects of CCR5 inhibitors on chemokine-induced cellular/immunological function are very complicated and clinical outcomes are difficult to predict, thus, careful long-term observation is needed for its clinical use.

Currently, MVC and enfuvirtide, an oligopeptide fusion inhibitor, are the only drugs that have been approved for clinical use as entry inhibitors. In addition, CVC, an oral, once-daily drug, was reported that it was effective and safe in the treatment of naïve HIV-1-infected subjects in a 48-week phase 2b trial^32^. However, most recent clinical trials of CVC are likely for the treatment of inflammation-related diseases such as NASH^13^. On the other hand, the development of other entry inhibitors, especially CXCR4 inhibitors, will also improve the utility of CCR5 inhibitors. In the present study, we have demonstrated pre-clinical efficacy and safety of newly reported potent CCR5 inhibitors with a different chemical structure from known CCR5 inhibitors. They also had different profiles in the CC-chemokine-binding inhibition or HIV-1 drug-resistance profile compared to those of MVC or APL. APL was dropped from clinical trials because of its severe hepatotoxicity, and such difference may result in a very different *in vivo* toxicity profile of GRL compounds. It is also important to develop more potent and metabolically stable CCR5 inhibitors with once-daily (QD) dosing regimens in order to complement the limitations of MVC in future.

In summary, the data generated in this study should help to design novel CCR5 inhibitors that are safe and active against all drug-resistant HIV-1s, which is very important as a countermeasure against possible occurrences of resistance to dolutegravir and other currently used anti-HIV drugs. Moreover, such detailed structural analysis may help us to understand the effects of chemokine receptor inhibitors on various immunological functions and pursue possible usages of them as immunomodulators or latent HIV-1 reversing agents.

## Methods

### Reagents

Three newly designed and synthesized CCR5 inhibitors, GRL-117C, GRL-10007C, and GRL-10018C (Fig. 1) are discussed in the present report. The methods for their synthesis and physicochemical profiles will be described elsewhere. The structures of these three compounds are shown in Fig. 1. A previously reported, spirodiketopiperazine (SDP) derivative, aplaviroc (APL) [4-[4-[(3 R)-1-butyl-3-[(1 R)cyclohexylhydroxymethyl]-2,5-dioxo-1,4,9-triazaspiro [5.5] undec-9 ylmethyl] phenoxy] benzoic acid hydrochloride]^16,33^, was used as a reference compound. Maraviroc (MVC), TAK-779, and SCH-C (SCH-351125) were synthesized as previously described^34–36^.

### Cells and viruses

MAGI-CCR5 cells^18^ were maintained in DMEM supplemented with 10% fetal calf serum (FCS: Gemini Bio-Products, West Sacramento, CA), 200 μg/ml G418, 100 μg/ml hygromycin B, and 100 μg/ml zeomycin. The Chinese hamster ovary (CHO) cells expressing CCR5^19^ were maintained in Ham’s F-12 medium (GIBCO-BRL, Rockville, MD) supplemented with 10% FCS, 50 U/ml penicillin, and 50 μg/ml streptomycin in the presence of 5 μg/ml blasticidin S hydrochloride. TZM-bl cells were obtained from the NIH AIDS Reagent Program, and were cultured in DMEM with 10% FCS. Peripheral blood mononuclear cells (PBMCs) were isolated from buffy coats of HIV-1 seronegative individuals, and were activated with 10 μg/ml phytohemagglutinin (PHA) prior to use^16^. A laboratory wild-type R5-HIV-1 strain (HIV-1_Ba-L_)^37^ was employed for drug susceptibility assays. AD101 (experimental CCR5 inhibitor) and VVC-resistant HIV clones^7,8^ were provided by Dr. John P. Moore of Cornell University for this study. The clones were propagated with activated PBMCs, and were used for the assays. A clinical HIV-1 strain, HIV-1_KP-5pc_, and an MVC-resistant isolate, HIV-1_KP-5mvcR_, were prepared and used for antiviral assays, as previously described^10,38^. The following reagents (plasmids) for T/F HIV-1 viruses^22,24^ were obtained through the NIH AIDS Reagent Program, Division of AIDS, NIAID, NIH: pCH040.c/2625 (cat# 11740), pCH106.c/2633 (cat# 11743), pTHRO.c/2626 (cat# 11745), and pCH058.c/2960 (cat# 11856), from Dr. John Kappes and Dr. Christina Ochsenbauer.

### Anti-HIV-1 assay

Antiviral assays using PHA-PBMCs (p24 assay) were conducted as previously reported^16,38^. In brief, PHA-PBMCs (1 × 10^6^/ml) were exposed to 50 TCID_50_ of HIV-1_Ba-L_ or drug-resistant HIV-1 infectious clones in the presence or absence of various concentrations of drugs; 10-fold serial dilutions were performed in 96-well microculture plates. The amount of p24 antigens produced by the cells was determined on day 7 in the culture via a commercially available ELISA kit (PerkinElmer). The p24 production level in drug-free control cell cultures was used as the reference. Drug concentrations that resulted in 50% reduction (IC_50_) in p24 antigen production were determined. Antiviral assays using MAGI-CCR5 cells (MAGI assay) were conducted as previously reported^16,39^. Single-round virus infection assays using TZM-bl cells infected with R5-HIV-1s (HIV-1_YU2_, HIV-1_KP-5pc_, and HIV-1_KP-5mvcR_)^10^ were also conducted as previously described^25^.

### Inhibition of chemokine binding to CCR5

The inhibition of the binding of ^125^I-labeled CC-chemokines ([^125^I] RANTES, [^125^I] MIP-1α, or [^125^I] MIP-1β) to CCR5 by various CCR5 inhibitors was measured as previously described^16^. Calcium flux inhibition assays were also performed with CCR5 inhibitors, and were carried out with the Fluo-4 Direct^TM^ calcium reagent (Invitrogen); assays were conducted as previously described with minor modifications^40^. In brief, CHO-CCR5 cells (5 × 10^5^ cells) were exposed to Fluo-4 Direct^TM^ calcium reagent for 60 min at 37 °C in RPMI containing 5% FCS. Cells were then incubated with the test compound for 30 min at varying concentrations. Subsequently, cells were exposed to either MIP-1α, MIP-1β, or RANTES at 200 ng/ml. Relative increases in cytosolic Ca^2+^ levels after chemokine exposure were determined with flow cytometry (FACSCalibur, BD Biosciences), and IC_50_ values of cytosolic Ca^2+^ mobilization (Ca^2+^ flux) were compared with the Ca^2+^ flux level in drug-free control samples.

### Structural modeling of interactions between CCR5 inhibitors and CCR5

Crystal structure of CCR5 reported by Tan *et al*. (PDB: 4MBS) was used as the template^20^, and structures of the CCR5-inhibitor complexes were defined. The ligands were built using the Maestro software, and were minimized with the MacroModel program using the OPLS2005 force field. CCR5-inhibitor complexes were obtained by docking the ligand structures to CCR5, using Glide. Software tools from Schrödinger were used to define the models (Schrödinger, LLC, New York, NY)^41,42^.
